# Surveillance for the safety and effectiveness of artemether-lumefantrine in patients with uncomplicated *Plasmodium falciparum* malaria in the USA: a descriptive analysis

**DOI:** 10.1186/s12936-015-0881-2

**Published:** 2015-09-17

**Authors:** Alyson M. Gray, Paul M. Arguin, Kamal Hamed

**Affiliations:** Centers for Disease Control and Prevention, 1600 Clifton Road NE, Mailstop A06, Atlanta, GA 30329-4027 USA; Novartis Pharmaceuticals Corporation, One Health Plaza, East Hanover, NJ 07936-1080 USA

**Keywords:** Artemether-lumefantrine, Effectiveness, Malaria, Safety

## Abstract

**Background:**

Data from clinical studies show that artemether-lumefantrine (AL) is effective and well tolerated in adults and children with uncomplicated *Plasmodium falciparum* malaria. However, data on effectiveness and safety of AL in patients in non-endemic settings are limited.

**Methods:**

A 5-year surveillance plan included all AL-treated adult and paediatric patients with confirmed or suspected *P.* *falciparum* malaria in the USA, as reported to the National Malaria Surveillance System at the Centers for Disease Control and Prevention. Descriptive analyses included demographics, baseline characteristics, clinical effectiveness, and safety. From May 2010 to April 2015, demographics and baseline characteristics were collected for 203 patients and safety data for 108 patients. Treatment effectiveness data at day 7 were collected for 117 patients and at day 28 for 98 patients.

**Results:**

The majority of patients were male (58.6 %), Black (62.6 %), non-Hispanic (92.6 %), and likely malaria non-immune (80.8 %). The median age was 32 (range 1–88) years and the median body mass index was 25.5 (range 13.8–42.4) kg/m^2^. All patients with effectiveness data had confirmed (n = 116) or suspected (n = 1) malaria. The overall cure rate for patients treated with AL was 91.5 % (95 % CI 84.8–95.8 %) at day 7 and 96.9 % (95 % CI 91.3–99.4 %) at day 28. Adverse events were reported in four (3.7 %) patients, and there were no new or unexpected safety signals.

**Conclusion:**

AL was effective and well tolerated in the treatment of likely non-immune patients with *P.* *falciparum* malaria.

## Background

Malaria is the most common febrile disease resulting in post-travel hospitalization, and artemisinin-based therapy is considered to be the fastest and most potent current anti-malarial treatment [1, 2]. Artemether-lumefantrine (AL) (Coartem^®^, Novartis Pharma AG, Basel, Switzerland) is a fixed-dose combination of 20 mg artemether and 120 mg lumefantrine. Both components are blood schizonticides with complementary pharmacokinetic profiles and dissimilar modes of action, thus providing synergistic anti-malarial activity [3–5]. Artemether is absorbed rapidly and has an elimination half-life of around 1 h. It clears most of the parasite biomass, providing fast resolution of symptoms during the acute treatment period. Longer acting lumefantrine, with a half-life of 3 to 6 days, has a variable absorption rate that improves when administered with fat and is more effective in the recovery phase, eliminating residual parasites. The efficacy and safety of AL has been established in adult and paediatric patients in clinical trials, mainly in malaria-endemic regions such as Southeast Asia [6–11], sub-Saharan Africa [12–20] and Latin America [21]. However, effectiveness and safety data for the treatment of uncomplicated malaria in non-endemic countries are limited.

In 2006, the World Health Organization initially recommended that artemisinin-based combination therapy (ACT) be used as first-line treatment for uncomplicated malaria [22]. Since then, AL has been widely adopted throughout malaria-endemic countries, especially in sub-Saharan Africa, as a first- or second-line treatment for uncomplicated *Plasmodium falciparum* malaria. Additionally, Coartem^®^ has also been registered in various non-endemic countries including the USA [5, 23, 24]. It received approval from the US Food and Drug Administration (FDA) in April 2009, becoming the first ACT available for the treatment of uncomplicated falciparum malaria in the USA [24], and is recommended by the US Centers for Disease Control and Prevention (CDC) as a treatment option for uncomplicated malaria. Other treatment options for uncomplicated falciparum malaria include atovaquone-proguanil; quinine sulfate plus either doxycycline, tetracycline or clindamycin; mefloquine; and chloroquine in the case of malaria acquired in areas with chloroquine-sensitive parasites [25].

In the context of the USA’s approval of Coartem^®^, the FDA required that Novartis conduct a descriptive surveillance project on the use of AL tablets in non-immune travellers as a post-marketing requirement. This project was conducted through collaboration between Novartis and CDC by which AL-treated malaria cases in the USA were captured in the National Malaria Surveillance System (NMSS). This report aims to describe the demographics, baseline characteristics, clinical effectiveness, and safety outcomes for patients treated with AL in both US and foreign residents.

## Methods

### Cases

Malaria is a mandatory reportable disease in the USA. Cases are reported by healthcare providers or laboratory staff to local and state health departments, and reports are in turn transmitted to CDC through the NMSS [26].

Any patient with malaria reported to CDC who had received AL treatment was included in this analysis. Patients who were diagnosed by microscopy or by polymerase chain reaction (PCR) were considered as confirmed cases, while those diagnosed by a rapid diagnostic test (RDT) or clinically with no (or missing) microscopy were considered as suspected cases. Both confirmed and suspected cases were included in all analyses. However, patients with a positive RDT result followed by negative microscopy or a clinical diagnosis only were considered as non-malaria cases (i.e., neither confirmed nor suspected) and were only included in the descriptive analyses of demographics, baseline characteristics and safety.

### Surveillance study design

Patients treated with AL for confirmed or clinically suspected malaria were reported to CDC as a routine public health surveillance activity. Data collection was planned for a total of 5 years. Information was captured using the CDC Malaria Case Surveillance Report form [27]. Contact with the treating physician was initiated by telephone either when the treating physician sought guidance on treatment of a patient with malaria or after the malaria case was passively reported. This report includes a descriptive analysis of data covering the period from May 2010 to April 2015.

### Surveillance study assessments

Patient demographic and baseline characteristics included age, gender, race, ethnicity, height, weight, and likely malaria immune status. Persons were considered to be likely semi-immune if they were recent immigrants from or residents of malaria-endemic countries who were visiting the USA when they were diagnosed with malaria. Residents of non-endemic countries and US travellers were considered likely non-immune. Clinical effectiveness was assessed in cases with available follow-up information on the Malaria Case Surveillance Report form. Resolution of clinical signs and symptoms after start of AL treatment was assessed with the following question: “Did all signs or symptoms of malaria resolve without any additional malaria treatment within 7 days after treatment start?” For patients with an answer ‘yes’ to this question, effectiveness at day 28 was assessed using the follow-up question: “Did the patient experience recurrence of signs or symptoms of malaria during the 4 weeks after starting malaria treatment?”

### Statistical analysis

Data analysis was not based on a specific a priori statistical hypothesis and was simply descriptive. Summary statistics were presented for quantitative variables, and counts and percentages were calculated for categorical data. For the day 7 and day 28 cure rates, the proportions of cured patients and two-sided 95 % confidence intervals were calculated using exact Pearson-Clopper limits [28]. The proportion of cured patients was based on all patients with available effectiveness information. Effectiveness data were further stratified by age (≤16 years, 17–64 years, ≥65 years), body mass index [(BMI) < 25 kg/m^2^, ≥25 kg/m^2^], likely malaria immune status, malaria species (*P.* *falciparum*, other, undetermined), and status of malaria diagnosis (confirmed, suspected).

## Results

**Fig. 1:**
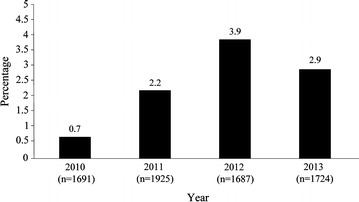
Percentage of confirmed malaria cases treated with artemether-lumefantrine in the USA by year (2010–2013)

A total of 203 AL-treated cases were reported from May 2010 to April 2015. Figure 1 shows the proportion of patients treated with AL per year based on the total number of confirmed malaria cases reported in the USA per year in the NMSS. All cases were imported (i.e., US travellers or foreign visitors), with 61.1 % of the malaria infections acquired in West Africa. The remaining 38.9 % of infections were acquired in East Africa, Central Africa, South Africa, the Caribbean, and South America. The majority of patients were aged 17–64 years, male, Black, non-Hispanic, and likely malaria non-immune. Some patients were missing information on variables such as height and weight (i.e., 94 patients did not have a recorded height and 71 patients did not have a recorded weight). Therefore, it was only possible to calculate the BMI for 107 patients (Table 1).

**Table 1:** Demographic and baseline characteristics

Characteristic	N = 203^a^
Age (years)	
Mean ± SD	34.2 ± 18.5
Median	32
Range	1–88
Age (years) category, n (%)	
≤16	32 (15.8)
17–64	157 (77.3)
≥65	14 (6.9)
Gender, n (%)	
Male	119 (58.6)
Female	84 (41.4)
Race, n (%)	
Black or African American	127 (62.6)
Caucasian	55 (27.1)
Asian	9 (4.4)
Native Hawaiian or Other Pacific Islander	2 (1.0)
American Indian or Alaska Native	1 (0.5)
Missing	9 (4.4)
Ethnicity, n (%)	
Hispanic or Latino	2 (1.0)
Not Hispanic or Latino	188 (92.6)
Missing	13 (6.4)
Height (cm), N = 109	
Mean ± SD	165.7 ± 21.8
Median	172.7
Range	80.0–198.1
Weight (kg), N = 132	
Mean ± SD	71.1 ± 23.8
Median	72.7
Range	9.0–144.1
BMI (kg/m^2^), N = 107	
Mean ± SD	25.6 ± 5.9
Median	25.5
Range	13.8–42.4
BMI (kg/m^2^) category, n (%)	
<25	50 (24.6)
≥25	57 (28.1)
Missing	96 (47.3)
Immune status, n (%)	
Likely non-immune	164 (80.8)
Likely semi-immune	39 (19.2)
Malaria species, n (%)	
*P. falciparum*	160 (78.8)
Other *Plasmodium* species (including *P. vivax*, *P. ovale*, *P. malariae*)	26 (12.8)
Undetermined	17 (8.4)
Malaria diagnosis, n (%)	
Confirmed by microscopy	195 (96.1)
Confirmed by PCR	6 (3.0)
Suspected	1 (0.5)
RDT positive (microscopy negative)	1 (0.5)

BMI, body mass index, calculated in kg/m^2^ as weight (in pounds) × 703/(height)^2^ (in inches); SD, standard deviation

^a^N = 203 unless indicated otherwise

Table 2 outlines day 7 and day 28 cure rates. One patient was excluded from the effectiveness analysis because the patient had a positive RDT followed by a negative microscopy. For the day 7 cure rate, effectiveness data were available for 117 of 202 patients with confirmed (n = 116) or suspected (n = 1) malaria due to *P.* *falciparum* or another species. For the day 28 cure rate, effectiveness data were available for 98 patients with confirmed malaria. Effectiveness results were analysed with missing effectiveness data excluded.

**Table 2:** Day 7 and day 28 cure rates

	Day 7 cure rate (N = 117)		Day 28 cure rate (N = 98)	
	n (%)	95 % CI	n (%)	95 % CI
Overall	107 (91.5)	84.8–95.8	95 (96.9)	91.3–99.4
By age (years)				
≤16	14/15 (93.3)	68.1–99.8	11/12 (91.7)	61.5-99.8
17–64	88/96 (91.7)	84.4–96.3	78/80 (97.5)	91.3–99.7
≥65	5/6 (83.3)	35.9–99.6	6/6 (100.0)	54.1–100.0
By BMI (kg/m^2^)				
<25	35/39 (89.7)	75.8–97.1	28/30 (93.3)	77.9–99.2
≥25	39/40 (97.5)	86.8–99.9	38/39 (97.4)	86.5–99.9
Unknown/not reported	33/38 (86.8)	71.9–95.6	29/29 (100.0)	88.1–100.0
By immune status				
Likely non-immune	89/99 (89.9)	82.2–95.1	78/81 (96.3)	89.6–99.2
Likely semi-immune	18/18 (100.0)	81.5–100.0	17/17 (100.0)	80.5–100.0
By malaria species				
*P.* *falciparum*	81/90 (90.0)	81.9–95.3	73/75 (97.3)	90.7–99.7
Other *Plasmodium* species (including *P.* *vivax*, *P.* *ovale*, *P.* *malariae*)	18/18 (100.0)	81.5–100.0	16/17 (94.1)	71.3–99.9
Undetermined	8/9 (88.9)	51.8–99.7	6/6 (100.0)	54.1–100.0
By status of malaria diagnosis				
Confirmed	106/116 (91.4)	84.7–95.8	95/98 (96.9)	91.3–99.4
Suspected	1/1 (100.0)	2.5–100.0	0/0 (0.0)	–

CI, confidence interval, calculated according to the exact Pearson-Clopper method

At day 7, the overall cure rate was 91.5 % (107/117). Out of the ten patients who did not resolve their malaria by day 7, seven had severe malaria at baseline and should not have received AL to begin with, one had an adverse event of nausea that was considered by the treating physician to be related to AL and was switched to atovaquone-proguanil, one was rehospitalized because of haemolytic anaemia thought to be due to persistent malaria and was discharged on quinine and doxycycline, and one did not complete the treatment course due to a supply shortage at the treating hospital. At day 28, clinical effectiveness data were available for 98 patients and the overall cure rate was 96.9 % (95/98). Three patients experienced a recurrence of signs or symptoms of malaria within 28 days. Of the 19 patients who were assessed at day 7 but not at day 28, data were missing for nine and were not collected for the ten patients who did not resolve at day 7 and were mainly switched to other anti-malarials. The day 7 (91.5 %) cure rate is not a true representation of the effectiveness of AL as at least seven patients should not have received AL for treatment of their malaria. Notably, although 22 other patients with severe malaria resolved upon receiving AL without further treatment, AL is indicated only for the treatment of uncomplicated falciparum malaria.

Adverse events (AEs) were reported in four (3.7 %) patients (Table 3), including three AEs (two anaemia and one nausea) suspected to be related to AL. Anaemia was reported as a serious adverse event (SAE) in two (1.9 %) patients. One had haemolytic anaemia and was rehospitalized, and the second received an outpatient blood transfusion due to ongoing haemolysis. Both of these SAEs were considered by the treating physicians to be related to ineffective treatment with AL, but there was no evidence of treatment failure in either case. Follow-up information was provided only for the first case. The patient experienced a haemoglobin decrease of 3 g/dl with a concurrent increase in lactate dehydrogenase and decrease in haptoglobin 12 days after the first dose of AL, which is consistent with the post-artemisinin delayed haemolysis syndrome [29]. Of note, this syndrome seems to occur in patients with hyperparasitaemia who should not be treated with oral therapy such as AL. The second patient was incorrectly diagnosed as having only falciparum malaria at baseline while the patient was actually co-infected with *Plasmodium* *vivax*. AL cleared the original infection, but one month later, after re-appearance of symptoms, the patient was treated with atovaquone-proguanil, doxycycline and primaquine, and the vivax co-infection was cleared.

**Table 3:** Adverse events and serious adverse events up to day 28

	Adverse events (N = 108)	Serious adverse events (N = 108)
	n (%)	
Anaemia	2 (1.9)	2 (1.9)
*P. vivax* malaria	1 (0.9)	–
Nausea	1 (0.9)	–

Cases are often reported to CDC long after they occurred or were originally notified (due to delays from the applicable state or treatment facility), and the lag time between occurrence of a case and its reporting to CDC can vary from a few days to 1 year or longer. Data available from 150 cases showed that they were reported to CDC on average 84 (range 1–733) days after the onset of signs and symptoms. As a result, there are possibly additional cases that may have been applicable to the timeframe of this project but were excluded due to a delay in reporting.

The delay in case reporting and retrospective data collection, small sample size, and incomplete data mainly because of loss to follow-up are all inherent limitations of this surveillance project. Other limitations include the non-comparative and non-randomized methods of data collection, unsupervised treatment, and clinical effectiveness and safety outcomes that were solely determined by the healthcare provider. Therefore, effectiveness data from this surveillance could hardly be considered as robust as those from some effectiveness studies conducted in endemic countries [15, 19]. Despite these limitations, such data represent the best available outcomes data for AL treatment in the USA and correlate well with results reported from other settings including those from prospective interventional studies.

## Conclusion

AL was effective and well tolerated in the treatment of likely non-immune patients in the USA with falciparum malaria.

## Abbreviations

AE: adverse event
ACT: artemisinin-based combination therapy
AL: artemether-lumefantrine
BMI: body mass index
CDC: Centers for Disease Control and Prevention
FDA: Food and Drug Administration
NMSS: National Malaria Surveillance System
PCR: polymerase chain reaction
RDT: rapid diagnostic test
SAE: severe adverse event
